# Extracellular Vesicles in Non-Small-Cell Lung Cancer: Functional Role and Involvement in Resistance to Targeted Treatment and Immunotherapy

**DOI:** 10.3390/cancers12010040

**Published:** 2019-12-21

**Authors:** Luigi Pasini, Paola Ulivi

**Affiliations:** Biosciences Laboratory, Istituto Scientifico Romagnolo per lo Studio e la Cura dei Tumori (IRST) IRCCS, 47014 Meldola, Italy; luigi.pasini@irst.emr.it

**Keywords:** extracellular vesicles, non-small cell lung cancer, resistance, liquid biopsy, diagnostic biomarkers

## Abstract

Targeted and immunological therapies have become the gold standard for a large portion of non-small cell lung cancer (NSCLC) patients by improving significantly clinical prognosis. However, resistance mechanisms inevitably develop after a first response, and almost all patients undergo progression. The knowledge of such a resistance mechanism is crucial to improving the efficacy of therapies. So far, monitoring therapy responses through liquid biopsy has been carried out mainly in terms of circulating tumor (ctDNA) analysis. However, other particles of tumor origin, such as extracellular vehicles (EVs) represent an emerging tool for the studying and monitoring of resistance mechanisms. EVs are now considered to be ubiquitous mediators of cell-to-cell communication, allowing cells to exchange biologically active cargoes that vary in response to the microenvironment and include proteins, metabolites, RNA species, and nucleic acids. Novel findings on the biogenesis and fate of these vesicles reveal their fundamental role in cancer progression, with foreseeable and not-far-to-come clinical applications in NSCLC.

## 1. Introduction

In the last ten years, treatment of non-small cell lung cancer (NSCLC) has been revolutionized by the introduction of targeted and immunological therapies in clinical practice [1,2,3]. Currently, the use of tyrosine kinase inhibitors (TKIs) against the epidermal growth factor receptor (EGFR) and the anaplastic lymphoma kinase (ALK) are the first treatment of choice for patients carrying alterations in the *EGFR* and *ALK* genes, as well as for patients with alterations in the proto-oncogene tyrosine-protein kinase ROS-1 (*ROS1*) [4]. On the other side, immune-checkpoint inhibitors represent the first treatment of choice for patients with high expression of the programmed cell death ligand 1 (PD-L1), as monotherapy (pembrolizumab), or in combination with chemotherapy for those patients presenting with no targeted alterations (nivolumab or atezolizumab) [4]. Although both types of treatment prolong progression free survival and improve patient prognosis, resistance mechanisms inevitably arise, leading to disease progression in almost all patients [5]. Currently, molecular profile of NSCLC is assessed by analyzing nucleic acids derived from the primary tumor tissue, but advancements in the ability to detect by longitudinal liquid biopsies the presence of tumor-released particles, including circulating tumor DNA (ctDNA), miRNA, and extracellular vesicles (EVs), has enabled clinicians to better understand the dynamic evolution of this disease [6].

EVs are defined as a heterogeneous group of membrane-delimited nanosized particles actively released by any cell type, including cancer cells [7]. A definite nomenclature consensus has not been established yet, and the various subtypes of cell-released vesicles are classified into exosomes, microvesicles, microparticles, ectosomes, oncosomes, apoptotic bodies (apoEVs), mostly based on their cellular origin and mechanism of biogenesis. However, the improvements of EV isolation methodologies and the emergence on specific markers of EV subtypes have supported the establishment of common guidelines to define experimentally the biological function of different EVs [8]. EVs can also be partially characterized by their size, which can range from 40–50 nm to over 1000 nm, and their molecular content, which can comprise various nucleic acid species, proteins, metabolites, or activated signaling molecules [8].

In this review, we will give particular attention to EVs of endosomal origin (defined as exosomes) and plasma membrane-derived microparticles (microvesicles) [9]. Exosomes and microvesicles share common sorting machineries, although they generate from different cellular compartments and it is the nature and the cellular abundance of the cargoes that determines the fate of a particular vesicle [10]. Several studies have demonstrated the functional relevance of EV release into circulation, indicating their major role in intercellular communication via transfer of their biological material to the recipient cells [10,11]. This evidence has led to the notion that EVs may support tumor growth as direct contributors to the initiation of oncogenesis, immunomodulation, metastatization, and resistance to therapy [12]. Many efforts have been made to understand EV function during cancer progression and, although current protocols of EV isolation are not effective in discriminating normal EVs from cancer-cell-derived EVs, thanks to most recent technical advances, we can envisage the introduction of EV-based liquid biopsy in the near future for the clinical practice of some cancers, including NSCLC.

## 2. Extracellular Vesicles Biogenesis and Fate

**Figure 1:**
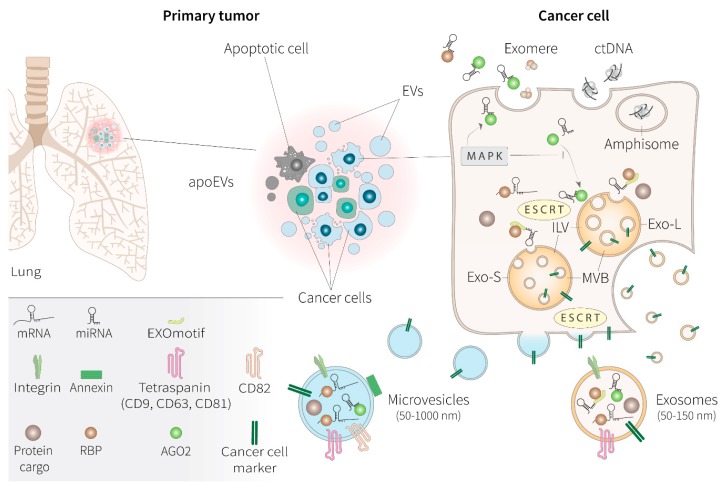
Biogenesis and heterogeneity of tumor-derived extracellular vesicles. A tumor mass is constituted of a heterogeneous population of cancer cells, constantly releasing a variety of extracellular vesicles (EVs), apoptotic bodies (apoEVs), and non-vesicular particles, including exomeres, all involved in the transport of nucleic acids and proteins that can have an active role in promoting tumor survival and aggressiveness. Cargo sorting into EVs involves the endosomal sorting complex required for transport (ESCRT) and partner proteins that include annexins (annexin A1) and tetraspanins (CD9, CD63, CD81, CD82). By reaching their destination exosome cargoes are sorted through the intraluminal vesicles (ILV), which form following the inward of endosomal membrane of the multivesicular bodies (MVBs) and are ultimately secreted upon fusion with the cell surface. Exosomes, similarly to microvesicles, display the same topology of membrane as the cell of origin and can potentially express cancer cell-specific markers. Microvesicles, however, generate from the outward budding of the plasma membrane. Different subsets of exosomes (large and small) with distinct molecular composition have been identified, along with the discovery of a population of abundant non-membranous nanoparticles involved in tumor growth, called exomeres. Exosomes are preferentially enriched with specific populations of miRNA, destined for exportation thanks to the presence of an EXOmotif, recognized by specialized RNA binding proteins (RBPs). During oncogenesis, activation of the mitogen-activated protein kinase (MAPK) pathway suppresses AGO2-mediated sorting of miRNAs to EVs, while favoring AGO2-independent EV loading of miRNAs and non-EV-associated miRNA release. Potentially, microvesicles can accommodate larger RNA species, including transcribing mRNAs. Nucleosomal double strand (dsDNA), although previously reported to be associated with exosomes, might not be directed to the ILVs, and it is instead released through EV-independent mechanisms involving intermediate organelles termed amphisomes. miRNA, microRNA; mRNA, messenger RNA; AGO-2, Argonaute 2; Exo-S, small exosome vesicles; Exo-L, large exosome vesicles.

EVs are a group of membranous structures that can be actively released by any cell, which are highly heterogeneous for their origins and content, and are considered to be ubiquitous mediators of intercellular communication [9]. Typically, EVs are classified based on their biological functions and the cellular compartment they originated from, rather than their size and membrane markers, which are normally overlapping between the different types of EVs [7]. In fact, the same marker can be displayed on different EV subtypes, although some markers can be enriched on the surface of particular vesicles, depending on the cell of origin [13]. The most established subtypes of EVs are exosomes, the size of which vary between 40 and 150 nm, and microvesicles that can be up to 1 μm [8,9]. In contrast to microvesicles, which fall from the cell surface due to budding of the plasma membrane, exosomes are derived from the endolysosomal pathway through the intraluminal budding of multivesicular bodies that eventually fuse with the cell membrane [10]. The molecular machineries involved in the different steps of EV biogenesis are for the most part common to exosomes and microvesicles, and the transmembrane proteins sorted on both types of EVs normally reflects the same topology as on the cell membrane (Figure 1).

Exosome cargoes reach their destination either from the Golgi or by internalization from the plasma membrane, before being sorted to the intraluminal vesicles (ILV) that are formed by the inward of endosomal membrane of the multivesicular bodies (MVBs) and are ultimately secreted upon fusion with the cell surface [14]. Thus, exosomes practically are ILVs with distinct biological functions related to their being secreted in the extracellular space [15]. In this context, syntenin is a key adaptor protein that links MVBs to the cytoskeleton, by regulating exosome biogenesis in concert with the ALG-2 interacting protein X (Alix) and the endosomal sorting complex required for transport (ESCRT), while acting as a potential regulator of endosomal targeting of exosome cargoes [16]. For example, selective recruitment of functional receptor proteins on exosomes, including integrin β1 or the major histocompatibility complex (MHC) class I molecules, is regulated at this level of exosome biogenesis [16]. Exosomes can also form in an ESCRT-independent manner that requires the involvement of tetraspanins, specifically CD63, the results of which are particularly enriched on the membrane of exosomes [17]. Tetraspanins, including also other members of the family, CD81 and CD9, directly regulate the selective intracellular routing of cytoplasmic molecules to ILVs, which can result in the co-sorting with other cytosolic proteins, such as the chaperone heat shock 70 kDa protein (HSP70), typically found in exosomes derived from most cell types [18]. Various RNA species, mostly including microRNAs (miRNAs) and small non-coding RNAs (ncRNAs), are transported by exosomes.

The presence of DNA inside EVs depends on methodological drawbacks that are not completely clear [19]. Short DNA sequences traditionally found after exosome isolation could be derived from the co-purification with these EVs during standard isolation methodologies. In fact, double stranded DNA might not be associated with exosomes or with any small EVs at all. DNA apparently is not directed to the ILVs, and it is instead released by the cell through independent mechanisms via intermediate organelles, originating from the nucleus and termed amphisomes, to be liberated, alongside EVs, in the extracellular as free nucleosomes [20].

After loading of their cargo, ILVs are targeted by the plasma membrane to be released as exosomes through the specific involvement of the ESCRT-1 component tumor susceptibility gene 101 protein (TSG101) and RAS-related protein (RAB) GTPases, while fusion with the cell membrane is guided by the soluble NSF attachment protein receptor (SNARE) complex [21,22].

Microvesicles follow a different route of biogenesis, which takes place at the level of discrete microdomains of lipids and membrane-associated proteins that cluster within the plasma membrane after the induction of local changes in Ca2+ concentration [23]. These events are accompanied by modifications of actin and myosin microfilaments that induce the lipid flipping while microvesicles are pinched off the plasma membrane [24]. Release of microvesicles involves TSG101 and ATP-dependent contraction. These molecular rearrangements of the plasma membrane is specifically driven by annexins, such as the annexin A1, along with the action of the tetraspanins CD9, CD81, and CD82 [10]. Microvesicles can be loaded with the same typology of cargos as exosomes through the ESCRT machinery, although microvesicles, given their larger size, might easily accommodate long mRNAs [10,20,25].

The fate of EVs depends on the specific composition of surface receptors that will target them to the right cell type. EVs can remain bound to the plasma membrane through tetraspanin-guided interaction with integrins while waiting for their cargo to be released, initiate signaling transduction, or engage the antigen presentation pathways [26]. EV fusion with recipient cells requires docking at the plasma membrane, followed by activation of extracellular receptors and vesicles internalization, which can be mediated either by clathrin-dependent or clathrin-independent endocytosis [27].

The interdependence of the intracellular machineries required for release and internalization of both exosomes and microvesicles facilitates the development of shared mechanism of deregulation that can be adopted by cancer cells to take over the secretome apparatus [28]. A tumor constantly releases EVs to alter the composition of the microenvironment and deliver the oncogenic signaling to target organs and modify the metastasis organotropism. Cancer EVs are able to modulate the immune response through MHC antigen presentation or trigger T cell apoptosis, influence the cross-talk with the surrounding stroma and induce fibroblast differentiation to eventually favor the migration of tumor cells through a permissive endothelium [29]. For example, patterns of integrin composition are used by cancer cells to target their exosomes to distinct organs and promote the premetastatic niche [30]. Cancer EVs normally have the same set of surface markers to those of healthy cells, being indistinguishable from their normal counterparts, although CD9 and CD81 overall seem to be more abundant, but mutually exclusive, on cancer EVs [25,31].

Apoptotic bodies are a less diffuse type of EVs, with a broad size range of 50 nm to 2 μm [32]. These EVs are released by dying cells, upon fragmentation of their plasma membrane. When derived from cancer cells, apoEVs are able to influence the phenotype of surrounding tumor cells to promote their survival and aggressiveness, through the transfer of spliceosome components that can alter mRNA splicing in recipient cells [33].

## 3. Content of Extracellular Vesicles and Functional Role in NSCLC

EVs are lipid-bilayer containers of a variety of biologically active signaling molecules, proteins, nucleic acids, functional lipids, and metabolites that are scheduled for secretion in the extracellular space in response to the microenvironment (Figure 1). The cargos associated with EVs can also reflect the pathological state of a cell. Vesiclepedia represents a consistent effort to create a compendium of EV contents, by experimental update from the biomedical community [7]. So far, this manually-curated database has provided constant annotation of the diversity of EV assortments, including the top 100 EV-associated proteins, data obtained from over 38 thousands RNA entries, and more than 600 metabolites identified in EVs.

Cargoes are considered the first regulators of EV formation, when the future fate of a particular vesicle is being determined [10]. Hence, potential cargoes that are enriched in the forming vesicle are targeted at the site of EV production, following a stepwise mechanisms that recruit sorting machineries that will be specific for what type of EVs are going to be produced, whether they are exosomes or microvesicles [27,34]. For example, augmented cellular expression of some small RNA species will probably stimulate the production of exosomes over microvesicles [25]. Size fractionation of small, medium, and large EVs, followed by RNA sequencing, indicated that larger-size or medium-size EVs reflects the protein-coding transcriptome, which is mostly loaded with mRNAs, whereas small EVs, like exosomes, are enriched by small non-coding RNAs [25]. Specifically, two subpopulations of exosomes (namely, small exosomes, Exo-S, and large exosomes, Exo-L) have been identified to be loaded with a different set of miRNAs [35]. These two separate subpopulations, besides having different cargo compositions, are also distinguishable by the superficial distribution of their proteoglycans, suggesting that they might have distinct biological functions and specific types of target cells [36]. Advancement in the isolation techniques have pointed out the fact that functionality of the different types of EVs is indeed mirrored by their composition, in contrast to traditional methods that yielded a mixture of vesicular and non-vesicular particles enriched in exosomes and almost free of larger EVs. Most significantly, contrary to what was generally previously thought, extracellular DNA is not associated with exosomes, but could instead be easily co-purified with the small EV fraction during standard isolation protocols while, revised high-resolution isolation protocols of density gradients followed by direct immunoaffinity capture of EVs, separate DNA together with the non-vesicular fraction [20]. This DNA is wrapped around histones, and it is possibly released by the cell alongside EVs or during apoptosis. Nevertheless, in the past decade, circulating DNA has been defined as the gold standard for clinical diagnosis by liquid biopsy in cancer, with particular relevance in NSCLC. In fact, although cytosolic DNA can also be released by active processes of secretory autophagy [20], it might not directly reflect the ongoing process of functional differentiation of a cell during oncogenesis or metastatization. The need to find dynamic markers of clinical utility has oriented efforts towards the study of miRNA expression and mutations on the mRNA molecules associated to EVs [6]. In particular, extracellular miRNAs can have a primary role in cell-to-cell communication, gene expression regulation, and cell reprogramming of target cells, as well as the exploration of the mechanisms of their release in the body fluids, assuming that an escalating focus on these molecules in the course of the last 2 to 3 years continues [37]. Out of total cell-free biofluid RNA, small non-coding RNA species, including miRNAs, are the most abundant in circulation, and are co-purified with both small EVs and non-EV particles [20,38]. A fraction of the functional miRNAs found into the blood are thought to be passively released by dying cells as complexes with RNA-binding proteins (RBPs) or encapsulated within apoptotic bodies [39,40]. Once outside in the extracellular space, free miRNA are only stable when bound to RBPs, like Argonaute2 (AGO2) [39]. Protein-RNA complexes are necessary to protect circulating miRNAs from plasma RNAases, and AGO2, the same effector of miRNA-mediated silencing [41], can also guide the release in the circulation of a bunch of cellular non-EV associated miRNAs with active biological functions [42]. Cell-released miRNAs can also be transported in the plasma and delivered to recipient cells by other non-vesicular particles, like high-density lipoprotein (HDL) complexes and exomeres [38,43]. However, most of the cell-released miRNAs involved in the direct silencing of cellular mRNAs are selectively delivered to target cells by loading within small EVs [20,38]. The miRNAs destined to be exported possess a conserved recognition motif, called the EXOmotif, which guides the binding to a series of specialized RBPs [44,45]. The selective binding of these RBPs is necessary for miRNA sorting into EVs, and coordinate the intracellular and extracellular miRNA distribution [46]. Hence, only a specific subpopulation of the total miRNAs are actively secreted outside the cell, and the relative abundance of miRNAs to be directed into the EVs is further controlled by dynamic post-transcriptional modifications [47]. Transcriptome profiling of EVs shows that miRNAs are actually a major component of the EV-RNA cargo, along with a minority of other small RNAs and protein-coding and long-noncoding transcripts [48]. Cancer-cell exosomes can even produce miRNAs cell-independently, by performing the entire process of miRNAs maturation from precursor miRNAs (pre-miRNAs), via their own Dicer and AGO2. These exosomes, when isolated from the blood of cancer patients, can alter the target cell transcriptome in a canonical Dicer-AGo2-dependent miRNA-mediated gene silencing, and promote oncogenic transformation of normal epithelial cells [49]. Single-EV analysis reveals that there is a strong heterogeneity of protein and RNA assortments among the EVs [25]. EVs that are in the exosome-range size are less heterogeneous in their protein content, compared to larger vesicles, but may be enriched in distinct miRNA populations [25,36]. Interestingly, cell-free non-vesicular ribonucleoprotein (RNP) particles also can work in the transport of small RNAs that are different from those contained in the exosomes, and these mostly include tRNAs [25].

Isolation of EVs from plasma of early stage NSCLC patients and subsequent miRNA-seq profiling, can define, with increased diagnostic accuracy, patient-specific subsets of miRNAs that are more expressed in lung cancer patients, compared with healthy individuals [50,51]. In fact, some miRNAs, which can work as strong biomarkers to predict disease progression, are selectively loaded into the EVs of NSCLC patients [52]. The composition of EV-incorporated miRNAs distinguish a fraction of miRNAs that is different from the cytoplasm pool, and can have a specific role in cancer development [46]. Classical kinase pathways can also regulate miRNA sorting into EVs to sustain cell proliferation of lung cancer cells [46]. The trafficking of potential oncogenic miRNAs between the cytosol and EVs is controlled by cellular signaling and the protein AGO2 is pivotal in determining which miRNA has to be sorted into EVs [42,53]. For example, during oncogenic activation of the mitogen-activated protein kinase (MAPK) pathway, downstream to mutant RAS GTPase, the interaction of AGO2-binding miRNAs with EVs is suppressed, while it favors AGO2-independent EV sorting of distinct subsets of miRNAs [54]. The majority of miRNAs with a functional role in cancer development are indeed loaded into exosomes, and cancer exosomes are specifically enriched in oncogenic miRNAs [25,49,55]. This property comes along with an empowered diagnostic potentiality when compared to whole-blood recovery miRNA profiles. Possibly, the miRNAs that are transported by EVs of cancer cells can directly prepare the metastatic niche by promoting drug resistance and immune escape. On the other hand, non-EV circulating miRNA might be released to follow different routes of regulation association with RNP for secretion, and provide a different contribution to the metastatic process. In this context, AGO2, which is a major regulator of miRNA activity, could be pivotal in orchestrating the interplay between the cellular localization and the EV-mediated extrusion of miRNA, while protecting those miRNAs that enter the circulation through passive mechanisms [39].

## 4. Role of Extracellular Vesicles in Drug Resistance

### 4.1. Immunomodulation, Resistance to Immune Checkpoint Inhibitors, and Premetastatic Niche Formation Caused by Extracellular Vesicles

Besides the functional delivering of their molecular content to recipient cells, EVs can be seen as signalosomes of several cellular processes, from the activation of cell surface receptor pathways or the transfer of membrane-associated molecules, including immunogenic antigens [56]. De facto, EV production originally evolved as a major communication mechanism during the regulation of the immune system. Numerous studies have shown that EVs can exert most of the functions as their parental cell by either working as primary effectors of T cell activation, with a direct role in antigen presentation and transfer of MHC antigen-bound molecules, or by inhibiting the immune cell function [57]. The first examples were EVs released by B cells and dendritic cells that were able to activate antigen presentation on T cells, inducing a specific immune response [58]. Soon after arrived the first demonstration showing that EVs can carry tumor-derived antigens and stimulate specific cytotoxic activity against the tumor [59]. EVs maintain the same topology of the antigen-presenting cell (APC) of origin, exposing the MHC-antigen complex at their surface. Optimal T cell activation occurs when EVs transfer the MHC-antigen complex in conjunction with the action of co-stimulatory molecules that are provided by the APC and are absent on the EV [60]. Classical tetraspanins, including the CD9, CD63, and CD81 are very important in the antigen recognition process [61]. Beyond their role in regulating EV formation, tetraspanins are pivotal in modulating immune-signaling complexes, as cell-surface co-stimulatory molecules, by facilitating the lateral positioning of MHC molecules and receptor-ligand interaction [62]. Co-engagement of CD81 and the antigen-receptor complex is fundamental for the activation of both T cells and B cells. Similarly, CD81 expressed on EVs derived from the same APC is involved in antigen presentation [63].

Induction of immunosuppression is intimately related to oncogenic progression through the development of adaptive mechanisms that bypass inhibitory action of proliferative pathways. In a context of active immune surveillance, there is a selective pressure for cancer cells to either downregulate neoantigen presentation or induce an immunosuppressive microenvironment [64]. In the past 15 years, there has been a drive to improve outcomes of NSCLC patients by shifting from traditional chemotherapy to advanced targeted therapies and immunotherapies. Specifically, most of the efforts have concentrated on the development of antibodies, like nivolumab, ipilimumab, and pembrolizumab, which could block the action of so-called immune checkpoint regulators [65]. Targeted immune checkpoints are the programmed cell death 1 (PD-1) and the cytotoxic T lymphocyte antigen 4 (CTLA-4), both expressed on T cells, while PD-L1 is produced by tumor cells [65].

During therapy, immune escape in NSCLC evolves through a multistep process visible at the level of DNA mutations in MHC genes, epigenome, and changes in RNA expression of immune regulatory genes [64,66]. Acquired resistance to immunotherapy by tumor cells is directly reflected in their EV production [67]. Most importantly, tumors release a continuous amount of immunomodulatory EVs, which can become massive during cytotoxic treatment [68]. Cancer cells can intensify their production of immunosuppressive EVs during the onset of resistance to anti-PDL1 immunotherapy [69]. Tumor-antigen-carrying EVs might be the vehicle by which the immune system spots the presence of cancer cells, and the parallel screening for antigenic EV could bring a great promise for the diagnosis of immune responsive tumors [67]. At the same time, tumor-derived EVs can be the conduit for immune surveillance escaping through cancer cells and for the failure of immunotherapies [70]. Cross-presentation of tumor-specific antigens by tumor-derived EVs can be downregulated, while suppression of T cell activation is often promoted through overexpression of PD-L1, which interacts with PD-1 receptor on T cells to elicit the immune checkpoint response. During treatment with immune checkpoint inhibitors, tumor cells evade immune response by upregulating the surface expression of PD-L1 and releasing EVs that expose PD-L1 to suppress the function of CD8 T cells and facilitate tumor growth in vivo [71]. Presence of interferon-γ (INF-γ) in the microenvironment stimulates the production of these immunosuppressive EVs and, in patients, circulating levels of EV PD-L1 that increase parallel to the levels of INF-γ, and can be used to stratify clinical responders to the PD-1 inhibitor pembrolizumab and non-responders [71]. PD-L1 is present on the surface of EVs isolated from the plasma of NSCLC patients, and the number of PD-L1-positive EVs correlates with PD-L1 expression level in the tumor tissue from the same patient [69]. PD-L1-expressing EVs that are derived from NSCLC cells can induce apoptosis of T lymphocytes, promoting tumor growth in mice. In this context, it seems that these EVs inhibit the secretion of INF-γ from Jurkat T cells to maintain a circuit of immune inactivation [69]. Tumor-derived EVs are also capable of inducing the expression of PD-L1 on monocytes, via transfer of tumor-cell specific noncoding RNAs, to facilitate immune escape accompanied by concurrent release of cytokines that contribute to cancer-related inflammation [72]. EVs that are isolated from the plasma of NSCLC patients can also contain the mRNA of PD-L1, and the number of copies of the vesicular PD-L1 mRNA per ml of blood associates with a response to the anti-PD-1 antibodies nivolumab and pembrolizumab [73]. The expression of PD-L1 mRNA in plasma EVs, collected before and after surgery of NSCLC patients with stage II to stage III tumors and from NSCLC stage IV patients collected before and after anti-PD-1/PD-L1 therapy, shows a high correlation with PD-L1 expression in the corresponding lung tumor tissues, decreasing significantly after surgery and after anti-PD-1/PD-L1 therapy [74].

Every tumor that has grown to a considerable size has also somehow escaped immune surveillance, which is the prerequisite for cancer cells to colonize distant sites. Tumor EVs might trigger adaptive immune responses or suppress inflammation, depending on the status of a particular immune cell, shaping in this way the evolution of metastases under the selective pressure of the immune system. A primary tumor can seed out immunosuppressive EVs to remotely prepare the metastatic niche for the homing of cancer cells. Fusion of EVs with the recipient plasma membrane is regulated by the presence of specific docking proteins, receptors, and integrins. Vesicles released by cancer cells that have evaded the immune system can be targeted to favorite organs and, given the integrin pattern they present on the surface, promote the premetastatic niche of the specific cancer-cell type from which they have originated [30,75]. Organotropic metastasis formation in the lung, for example, is promoted by expression on the EV surface of the integrin α6β4, while EVs displaying the integrin αVβ5 are directed to the liver [30]. Once seeded at the metastatic niche, tumor EVs may engage the interferon pathway of normal cells, allowing further uptake of malignant EVs [76]. Subsequent internalization and delivery of internal cargos are typically followed by activation of intracellular signaling, which could be pivotal in determining the oncogenic transformation of target cells, and make the organ site hospitable to implantation and growth of cancer cells from the primary tumor [23,77]. Tumor-cell EVs are able to induce oncogenic transformation and anchorage-independent growth of healthy fibroblasts by carrying fibronectin or laminin that alter the intracellular signaling of membrane integrins [23]. The distant microenvironment can be further influenced by the release of immunosuppressive cytokines caused by tumor-derived EV [67].

**Figure 2:**
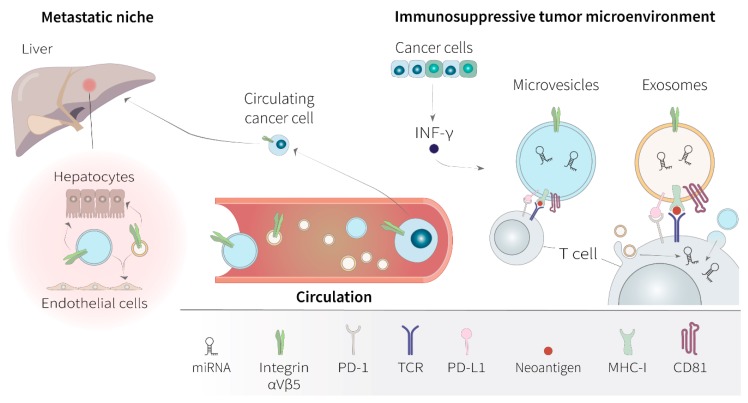
Functional relevance of extracellular vesicles in the oncogenesis of non-small cell lung cancer. In the contexts of non-small cell lung cancer (NSCLC), extracellular vesicles (EV)s, including both microvesicles and exosomes, can be regarded as cell signalosomes. EVs are able to influence the tumor microenvironment and modulate the immune response through downregulation of the T-cell receptor (TCR) activation by the programmed cell death ligand 1 (PD-L1), during neoantigen presentation via the major histocompatibility complex of class I (MHC-I) and co-engagement of the tetraspanin CD81. Production of immunosuppressive EVs in NSCLC patients is favored by circulating interferon-γ (INF-γ) in the tumor microenvironment. Tumor-derived EVs can display specific integrin patterns that target them to favorite organs, where they can influence the organotropism of the premetastatic niche. For example, organotropic metastasis formation of cancer cells in the liver is promoted by EV-expression of αVβ5 integrins. EV cross-talk with the surrounding stroma enables the endothelium to be more permissive for the extravasation of circulating cancer cells to further promote organ-specific invasion. Besides, delivery of the intraluminal microRNA (miRNA) EV cargo into recipient T cells, by membrane fusion or endocytosis, can have a major role in suppressing the anti-tumor inflammatory response.

The metastatic propensity of a primary tumor can also be inferred by the analysis of the vesicular RNA cargo [12]. A multitude of miRNAs that are transferred via EVs have been shown to have a potential role in anti-tumor immunity [78]. These miRNAs can exert key immune-modulatory and pro-oncogenic functions by targeting the mRNAs of T cell receptors [79], or by downregulating the expression of genes involved in the inflammatory response [80]. Moreover, miRNAs transported by tumor-derived EVs, like those secreted by lung cancer cells in hypoxic conditions, can induce infiltration of pro-tumorigenic macrophages via transfer of miR-103a that lead to subsequent downregulation of the phosphatase and tensin homolog (PTEN) mRNA levels [81]. Recently, characteristic EV-miRNA signatures of cancer-related inflammation have been associated with prediction of response to anti PD-1/PD-L1 therapy in NSCLC [82]. Hence, tumor-derived EVs can prepare the organ-specific metastatization of the cancer cell type they have originated from by inducing local and distant suppression of the immune system function, and expression profiling of integrins on the EV surface might reflect metastatization propensity to preferential organs. As well as being a carrier of antigens and immunosuppressive molecules, tissue-targeted EVs also constitute a mechanism of horizontal propagation of RNA-delivered information from APC to immune system cells (Figure 2).

### 4.2. Involvement of Extracellular Vesicles in Resistance to Targeted Therapy

With the increasing knowledge of molecular biology and genetics of tumors, the research and clinical application of targeted therapy has become a hot topic, with the aim of improving prognosis and guiding therapeutic decision by understanding the emerging of therapeutic resistance [5]. Each single lung cancer is characterized by an average of 300 DNA mutations, while only a few genes are able to promote tumorigenesis [83]. The two main NSCLC drivers are EGFR and ALK, along with ROS1 and the met proto-oncogene (c-MET), and other genes that are less frequently altered [4,84]. EGFR tyrosine kinase inhibitors (TKIs), including gefitinib, erlotinib, afatinib, and osimertinib, typically improve time to progression, response rates, and overall survival in EGFR-mutated patients, but acquired resistance to anti-EGFR TKIs is inevitable [85]. For this reason, improvement of the most effective diagnostic tools is necessary to predict the risk of developing drug resistance and ameliorate the clinical management of NSCLC patients. The clonogenic selection of the T790M mutation in the EGFR gene is the most frequent and well-known mechanism of resistance. Many studies have demonstrated the possibility to detect the T790M mutation on circulating tumor DNA (ctDNA) liquid biopsy, although the degree of sensitivity of such methodologies is quite low [86,87]. Similarly, after treatment of ALK-mutated patients with the drugs most commonly used at present, like crizotinib, ceritinib, and alectinib, a series of on-target and off-target resistance mutations may occur, which ctDNA analysis is generally insufficient for detecting [88]. Hence, analysis of tumor-derived EVs has emerged as an alternative approach to ctDNA to evaluate therapeutic effect of targeted therapy. Specifically, it has been reported that exosomal RNA can be a useful biomarker for detecting development of the T790M and other activating EGFR mutations, increasing sensitivity and specificity of traditional ctDNA analysis [89,90]. The importance of tumor EVs as clinical biomarkers is supported by the fact that EVs are directly implicated in the propagation of resistance mechanisms between cancer cells. Recently, the EXTRA study was launched with the aim to identify novel predictive biomarkers of resistance to afatinib by analyzing circulating and encapsulated biomarkers in peripheral blood [91]. EVs can be a vehicle to the onset of therapeutic resistance through the transfer of oncogenic miRNAs. For example, gefitinib-resistant cell lines are able to release exosomes enriched in specific miRNAs that are able to confer resistance phenotypes to recipient cells. In particular, the miR-214, the lncRNA H19, and the miR-21 are associated with gefitinib resistance [92,93,94], while EVs containing the lncRNA RP11 838N2.4 can propagate resistance to erlotinib [95]. ALK-rearranged cancer cells can also transfer the resistance to anti-ALK TKIs by releasing EVs containing a subset of specific miRNAs. In fact, EV-RNA profiling reveals that miR-21-5p, miR-486-3p, lncRNAs MEG3, and XIST are differentially expressed in the EVs secreted by the resistant subclones, and the circulating levels of these EV-associated miRNAs correlate with disease progression of EML4-ALK-translocated lung adenocarcinoma patients treated with ALK-TKIs [96].

EVs, found in the circulation of cancer patients refractory to therapy, can also transport nucleic acids molecules of wild-type EGFR and mutated EGFR that reflect the genetic signature of the original tumor [97]. However, it is know that cancer cell-derived EVs can reprogram quiescent cells towards a pro-tumorigenic phenotype more efficiently by direct transfer of cellular oncogenic protein kinases [98]. Cancer cells use EVs to transfer oncogenic ALK to normal cell in the surrounding tumor microenvironment and confer resistance to neighboring drug-sensitive cancer cells, via activation of the MAPK pathway [99]. Likewise, NSCLC cells can modify the function of adjacent cells by the exchange of EVs that transport constitutively-activated EGFR [100]. Interestingly, transduced EGFR translocate into the nucleus by a mechanism that is dependent on EV fusion with the nuclear envelope, rather than specific nuclear localization signals [101]. In the nucleus, EGFR is capable to activate pathways that are associated with tumor resistance to therapy-induced DNA damage and anti-EGFR treatment, in a mechanism that is independent of its conventional signaling from the plasma membrane [102,103]. Malignant cells can also share EVs that contribute to horizontal transfer of EGFR oncogenic isoforms, such the EGFRvIII, found in most aggressive tumors, and induce oncogenic activity downstream to MAPK and morphological progression towards a more invasive phenotype [97,104]. From the clinical perspective, detection of circulating EGFR protein from NSCLC patients would have important implications in defining reliable biomarkers of clinical prediction of anti-EGFR therapy [105]. Now, information derived from patients’ liquid biopsy might be finally complemented with the most advanced techniques of EV purification, along with accurate analysis of EV protein content nucleic acid [106].

## 5. Detection of Extracellular Vesicles and Their Application as Clinical Biomarkers for NSCLC

Over the past five years, explorative approaches for integrating liquid biopsy into the management of most common cancer types have been developed at a rapid rate [107], with over 360 clinical trials going on, including 32 studies focusing on EVs, of which more than 10% focus on lung cancer (https://clinicaltrials.gov/). Liquid biopsy of cancer patients can provide a comprehensive overview of tumor heterogeneity to help with diagnosis and guide patient care. Regulatory agencies in Europe and the United States have approved liquid biopsy tests in clinical routine for the detection of EGFR mutations on ctDNA in plasma of patients with NSCLC [108], with the aim to expand the diagnostic application of liquid biopsy to other NSCLC oncogenes, including ALK and ROS1 [87]. Although the analysis of ctDNA is currently the gold standard for routine diagnostics of NSCLC, the analysis of circulating EVs has emerged as a potential complementary methodology for implementing the detection sensitivity of drug-resistance mutations and tracking of tumor-specific alterations during disease progression and in the course of therapy [109].

Despite the substantial technical advances, the complexity associated with the enrichment of single or multiple EV subtypes with different composition poses continuous challenges to avoiding method-dependent bias. Methods for isolation from body fluids and analysis of EVs comprise separation on microfluidics, by density gradients, or through size exclusion chromatography, which can be followed by immunoprecipitation for further enrichment of specific subpopulations of EVs [110,111,112]. Combination of western blot, nanoparticle tracking, and flow cytometry is usually used to verify EV purity, and can be completed by using proteomics, lipidomics, RNA/DNA analysis to study EV composition [110]. The scientific community has agreed on the urgent need to standardize the procedure of EV isolation from patients’ liquid biopsies, validate EV-associated biomarkers, and put experimental guidelines into practice [7,113,114]. Moreover, highly stringent and novel isolation methodologies now provide a reappraisal of what classifies the variety of circulating nanoparticles, distinguishing vesicular and non-vesicular components, rather than exosomes or larger microvesicles [20]. The study of the functionality of these revised subpopulations avoided many controversies derived from non-specific isolation techniques and raised further interest in using selected subpopulations of EVs for successful biomarker analysis. However, EV analysis would be most beneficial in diagnostics if the procedure can specifically identify which antigens should be targeted in order to separate cancer-derived EVs from normal host cell-derived EVs.

Clinical application of EV-based diagnostics in NSCLC can be particularly helpful in the detection of oncogene mutations that confer sensitivity to specific drugs or are selected in those patients that develop pharmacological resistance. Tumor-released EVs can also help the early detection of clones that will develop immune escape through the overexpression of PD-L1 or the production of immunosuppressive miRNAs. Moreover, by defining tumor-specific miRNA signatures, EV content analysis can provide direct information on the dynamic transformation of a tumor mass and its clonal outgrowth [38,115]. Composition of small RNA species varies a lot between the human fluids, with a relative abundance of miRNAs in the blood plasma [116]. Mapping the diversity of extracellular RNAs (exRNAs), based on the use of specific RNA isolation methods, has revealed the importance of the related carriers. In fact, the accurate separation of exosomes and microvesicles from non-EV particles can facilitate the use of exRNA, which are present at variable concentrations depending on the body fluid analyzed, as powerful biomarkers [38,40]. Experimental data clearly show that most commercial kits preferentially enrich AGO2-bound miRNAs and only partially or not at all enrich EVs, whereas there is a need to apply methodologies that pre-isolate EVs prior to RNA extraction to specifically identify EV-associated exRNA [40]. Based on the evidence that blood levels of EV miRNAs significantly increases during cancer conditions, EV-associated miRNAs are now considered to be a most promising biomarker for the blood-based early detection of cancer [117]. In fact, when included into EVs, cell released miRNAs are rather stable and easy-to-detect [118], and, although most of the analysis on circulating miRNA is still exploratory, blood-based RNA profiling of cancer patients has provided a valuable source of information to understand tumor’s evolution and adaptation to therapeutic pressure in many clinical studies [114].

Compared to exosomes, which clearly show an enrichment of specific miRNA contents that are distinct from the cellular pool, microvesicles more closely reflect the source cell transcriptome, being particularly enriched in mRNA sequences [25]. Larger vesicles can function as shuttles between two cells for the transfer of protein-translating mRNAs [11,119]. These mRNAs could become a complementary genetic material of easy access for molecular diagnostics of oncogene mutations. For example, analysis of vesicles-associated nucleic acids for common B-Raf proto-oncogene (BRAF), KRAS Proto-Oncogene (KRAS), and epidermal growth factor receptor (EGFR) mutations has shown higher sensitivity compared to liquid biopsies of plasma ctDNA in association to clinical outcomes of patients with NSCLC [120]. EVs isolated from plasma of NSCLC patients can be used for EGFR genotyping through sequential liquid biopsies during treatment for the detection drug-resistance mutations, like the pT790M, demonstrating improved concordance with testing of tumor tissue compared to a conventional liquid biopsy of ctDNA [121].

To date, most available protocols to recover EVs from patients’ blood resulted in the recovery of a heterogeneous population of vesicles of uncertain origin, but the implementation of more robust technologies have facilitated the isolation of specific subpopulations of as few as ten EVs per microliter of plasma with direct impact on the performance of revealing predictive biomarkers [111]. Novel approaches have for the first time enabled the identification of cancer-derived EVs through a specific immunocapture method that targets membrane proteins of the tumor tissue of origin, separating cancer EVs from the bulk of background blood EVs [122,123]. Selective isolation of tumor-associated EVs can also be coupled with direct PCR-based quantification of oncogenic mRNAs following nanodroplet encapsulation of plasma EVs, and dramatically improve the sensitivity potential of liquid biopsy of BRAF and KRAS mutations [123].

**Figure 3:**
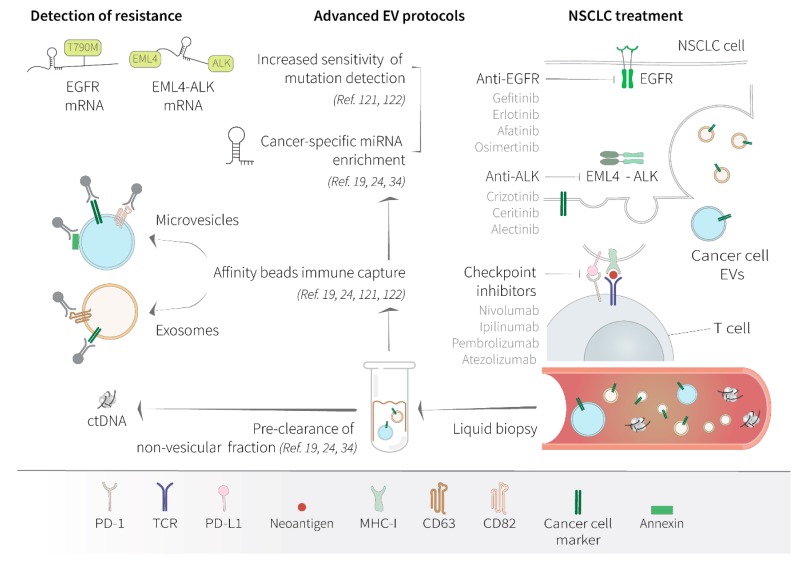
Significance of cancer cell-derived extracellular vesicles as biomarkers of drug resistance in non-small cell lung cancer. Conventional therapies of non-small cell lung cancer (NSCLC), either implying targeted tyrosine kinase inhibitors (TKIs) or anti-checkpoint antibodies, inevitably place the tumor cells under sustained selective pressure that eventually bring to pharmaceutical resistance in patients. Clonal evolution of drug-resistant cells can be captured by liquid biopsy and the data obtained from dynamic monitoring of tumor changes can be used to guide further therapeutic decisions. Clonal selection can be gene specific, by occurring through hotspot mutations in the therapy-targeted genes, for example in the anaplastic lymphoma kinase (ALK), typically fused with the echinoderm microtubule-associated protein-like 4 (EML4), and in the epidermal growth factor receptor (EGFR). Resistance to treatment in NSCLC can also be mediated by overexpression of the programmed cell death 1 (PD-1) receptor and its ligand, the programmed cell death ligand 1 (PD-L1), to elicit the immune checkpoint response. Currently, the molecular profile of lung cancers in liquid biopsy is obtained by using circulating tumor DNA (ctDNA) as a primary tumor marker. However, substantial improvements of the molecular approaches have been made to assess tumor heterogeneity through the analysis of tumor-released extracellular vesicles (EVs) from liquid biopsy. Revised differential separation protocols are often based on advanced microfluidics, immune capture of specific EV tetraspanins, affinity beads, and EV enrichment by antibody-based selection of surface cancer markers. Specific miRNA cargo can be enriched in subtypes of EVs with distinct biological functions and can be potentially used as biomarkers of oncogenic progression. For example, analysis of EV-associated mRNA might be particularly advantageous for the detection of mutations, like the substitution T790M in resistant NSCLC patients, and gene fusions, like the EML4-ALK in NSCLC, as well as splicing variants and changes in gene-expression profiles. In comparison to ctDNA molecules, which normally present two copies for cancer cell of origin, mRNA likely yields from highly expressed genes, occurring in thousands of copies per cells, and can be shed into the circulation within EVs at massive concentrations. At the moment most of EV-based analysis of circulating miRNA and mRNA remain exploratory and necessitate further clinical validation. miRNA, microRNA; mRNA, messenger RNA; TCR, T-cell receptor; MHC, major histocompatibility complex.

As a vehicle of extracellular RNA, EVs could be a considered as an easily accessible reservoir of biomarkers discovery (Figure 3) and molecular analysis of the different EV contents can provide complementary clinical information (Table 1).

Improvement of efficient and cost-effective methodologies for separation of exosomes and microvesicles population from the background of non-EV particles, based on their surface proteome composition, can foster the ultrasensitive detection of cancer-specific mutations on mRNA that could implement classical ctDNA blood analysis [36,124]. At the same time, construction of reference collections of vesicular RNAs that include predictive biomarkers for immunotherapy and targeted therapy will serve as a shared fundamental resource for future application of exRNAs [114,125].

## 6. Conclusions

In the wake of assigning an integrative view of the heterogeneity of resistance mechanisms, it is mandatory for to have an implementation of protocols that can assess the ongoing patient mutational status almost real time for the allocation of the specific therapies. Liquid biopsy poses as the best choice for the non-invasive monitoring of emerging resistance mechanisms at different time points, (i.e., at baseline and during treatment), which cannot be captured by conventional tissue biopsy. In most cases, measurement of ctDNA has permitted to recover substantial information about the onset of resistance mutations in NSCLC, even much before clinical progression. To achieve the highest grade of sensitivity, however, it is understandable that the measurement of ctDNA must be incorporated with more deviceful techniques. The discovery of the ubiquitous presence of EVs in the body fluids, coupled with the development of ever more reliable and low-cost technologies for the isolation of EVs and the implementation in the purification of their content promise to provide a substantial improvement in the detection of tumor markers, from routine blood draws. Emission of immunosuppressive organ-specific EVs from the primary tumor can be a precondition to prepare distant sites for metastatic invasion, and sensitive methodologies for tumor EV isolation and analysis can constitute a new frontier of cancer diagnostic to predict the metastatic spread and therapy outcome. Several challenges should be overcome for a possible implementation of EVs in the clinical practice, with the first and foremost challenge being the standardization of the methodologies for EVs isolation and purification from plasma. These methodologies should be easily usable by molecular diagnostic laboratories, with a low turnaround time and a low cost.

## Figures and Tables

**Table 1:** Comparison of the main biomarkers derived from extracellular vesicles for clinical application in non-small cell lung cancer.

EV Content	Potential Utility as NSCLC Biomarker
mRNA	gene expression, driving/resistance mutations, splice variants, gene fusions, tumor heterogeneity
miRNA	immune-suppressive signature
lncRNA	drug-resistance response
Protein	PD-L1 expression, neoantigen expression, patient MHC haplotyping

EV, extracellular vesicle; NSCLC, non-small cell lung cancer; mRNA, messenger RNA; miRNA, microRNA; lncRNA, long non-coding RNA; PD-L1, programmed cell death ligand 1; MHC, major histocompatibility complex.
